# Associated factors of nipple fissures in early postpartum women: A retrospective case-control study

**DOI:** 10.1097/MD.0000000000048125

**Published:** 2026-03-20

**Authors:** Lina Yang, Yang Li, Hui Yang, Hongmei Li, Junting Zheng, Yajing Zhang

**Affiliations:** a Department of Obstetrics and Gynecology, Hebei Medical University Third Hospital, Shijiazhuang City, China.

**Keywords:** associated factors, breastfeeding, case-control study, latch, nipple fissures, nipple pain, postpartum period

## Abstract

Breastfeeding-associated nipple trauma can compromise feeding effectiveness and contribute to early discontinuation. This single-center retrospective case-control study enrolled breastfeeding mothers who attended routine lactation consultation visits at our institution between June 2022 and June 2025, including 56 mothers with clinically documented nipple fissures and 56 controls without fissures. Maternal demographic, obstetric, infant, and feeding-related variables were extracted from electronic medical records and standardized lactation assessment forms. Analyses were conducted using IBM SPSS Statistics version 26.0 (IBM Corp., Armonk). Continuous variables are reported as mean ± standard deviation or median (interquartile range ), and categorical variables as n (%). Group comparisons used the Welch *t* test, Mann–Whitney *U* test, chi-square test, or Fisher exact test as appropriate. Univariate and multivariable logistic regression models were used to identify factors associated with nipple fissures, reporting odds ratios (ORs) and adjusted odds ratios (aORs) with 95% confidence intervals (CIs). Baseline characteristics were largely comparable; postpartum weight change differed between groups (*P* = .011). Mothers with nipple fissures reported less exclusive breastfeeding (*P* = .005), shorter feeding duration (*P* < .001), and more frequent poor latch and pain during suckling (both *P* < .001). In univariate analyses, exclusive breastfeeding and longer feeding duration were associated with lower odds of fissures, whereas poor latch, pain, and early bottle introduction were associated with higher odds. In multivariable analysis, shorter feeding duration (per 5-minute increase: aOR = 0.510, 95% CI 0.301–0.863; *P* = .012), pain during suckling (aOR = 3.826, 95% CI 1.143–12.808; *P* = .030), and early bottle introduction (aOR = 3.063, 95% CI 1.020–9.192; *P* = .046) remained independently associated with the presence of nipple fissures; however, given the retrospective design, these findings should be interpreted as associations rather than causal relationships. The retrospective single-center design and potential temporal ambiguity limit causal inference.

## 1. Introduction

Breastfeeding is recommended because it is associated with benefits for maternal and infant health; however, early breastfeeding difficulties remain common and are a frequent reason for early discontinuation. Recent clinical evidence continues to identify nipple pain and nipple trauma as prevalent postpartum problems that can impair milk transfer, reduce maternal confidence, and prompt supplementation practices that may further complicate breastfeeding.^[1]^ In a recent hospital-based evaluation of breastfeeding problems, nipple-related symptoms (including sore, wounded, or cracked nipples) were reported in a substantial proportion of mothers during the early postnatal period, underscoring the ongoing clinical burden of nipple injury.^[2]^ Nipple fissures represent a clinically relevant form of nipple trauma, typically characterized by linear cracks or splits in the nipple epithelium that may be accompanied by pain, bleeding, or surface disruption. Contemporary reviews describe nipple trauma as a spectrum that includes fissures, erosions, and wounds, and emphasize that untreated tissue disruption may contribute to pain persistence and breastfeeding cessation.^[3]^ Mechanistically, excessive compressive forces and shear stress concentrated on the nipple – often arising from shallow latch or suboptimal positioning – are consistently implicated in the initiation of nipple damage. Recent observational work analyzing early nipple damage patterns similarly highlights latch-related factors as proximal contributors to nipple injury in breastfeeding women.^[4,5]^ It is important to distinguish nipple pain, which may occur without visible tissue disruption, from nipple fissures, which represent clinically observable epidermal breaks.

Clinical management of nipple fissures frequently involves both correction of breastfeeding technique and symptomatic measures. Evidence syntheses published in the last several years have evaluated nonpharmacological topical approaches for breastfeeding-related nipple pain, reflecting the persistence of this clinical problem and the need for pragmatic, evidence-informed interventions.^[6]^ Individual trials have also examined specific topical agents (e.g., aloe-based treatments) in relation to pain reduction and healing among lactating women with nipple trauma.^[7]^ In parallel, device- and practice-related exposures warrant consideration. Breast pumping is widely used, and studies assessing comfort during expression report frequent nipple soreness, suggesting that pumping experiences and breastfeeding initiation problems may interact in ways that influence nipple symptoms.^[8]^ Moreover, equipment fit and settings may affect comfort; recent research on flange sizing indicates that sizing approaches can influence comfort outcomes during expression. Nipple shields are another commonly used aid, and recent data from maternity-ward settings suggest that early nipple shield use may be associated with interruption of exclusive breastfeeding before 6 months.^[9,10]^

Although previous studies have examined nipple pain and breastfeeding difficulties, fewer investigations have simultaneously evaluated maternal, infant, and feeding-related variables within a unified multivariable framework using real-world clinical records in early postpartum women. Furthermore, the distinction between nipple pain and clinically documented nipple fissures has not always been clearly maintained in prior literature.

Because breastfeeding success is also shaped by access to counseling and follow-up, recent guidance has continued to support behavioral counseling interventions to improve breastfeeding outcomes in postpartum populations. In addition, recent longitudinal work examining breastfeeding continuation has identified pain- and difficulty-related factors (including cracked nipples) among reasons associated with cessation.^[11]^ Against this background, further characterization of maternal, infant, and feeding-related factors associated with nipple fissures may help refine early clinical assessment and prioritize modifiable contributors within routine postpartum care. We hypothesized that feeding-related mechanical factors and early supplementation practices would be associated with clinically documented nipple fissures after adjustment for maternal and infant characteristics.

## 2. Methods

### 2.1. Study design

This study was approved by the Ethics Committee of Hebei Medical University Third Hospital. This retrospective case-control study enrolled breastfeeding mothers who attended our institution between June 2022 and June 2025. Participants were classified into a nipple fissures group (cases; n = 56) and a control group without nipple fissures during the same period (n = 56), with grouping determined based on documented clinical diagnoses and breastfeeding-related physical examination records. Eligible mothers were required to be actively breastfeeding during the postpartum period, have complete medical and lactation consultation records sufficient for risk factor assessment, and have a clear documented status regarding the presence or absence of nipple fissures; for controls, absence of nipple fissures throughout the corresponding observation window was confirmed by clinical documentation. Exclusion criteria included preexisting nipple or breast dermatoses, suspected or confirmed breast/nipple infection at baseline that could confound fissure assessment, history of breast or nipple surgery, major systemic diseases or medication use affecting skin integrity or wound healing, mixed or non-breastfeeding feeding patterns precluding accurate breastfeeding exposure characterization, and incomplete or missing key covariate data. The study was conducted in accordance with the Declaration of Helsinki and was approved by the institutional medical ethics committee; written informed consent was obtained from all participants for the use of their clinical data for research purposes. All exposure variables were extracted from documentation recorded at the index lactation consultation visit. Because data were collected retrospectively from routine clinical assessments, precise temporal sequencing between some exposures and the onset of nipple fissures could not be definitively established. Participants were consecutively identified from eligible lactation consultation records during the study period to minimize selection bias.

### 2.2. Diagnostic criteria for nipple fissures

In this study, nipple fissures were defined using an operational clinical criterion as a visible crack, split, or break in the epidermis of the nipple (with or without areolar involvement) documented on physical examination and/or lactation consultation records. This finding could be accompanied by nipple pain during feeding and/or superficial bleeding, exudation, or crusting, but these symptoms were considered supportive rather than mandatory for case ascertainment. This definition is consistent with commonly used clinical descriptions of nipple fissure/cracked nipple as small cracks or tears in the nipple skin that may bleed. For standardized coding (when applicable), the diagnosis corresponded to International Classification of Diseases, 10th Revision category N64.0 (fissure and fistula of nipple). To minimize misclassification, cases were not assigned when records described nipple soreness/erythema without skin disruption, and mothers with alternative primary diagnoses that could mimic fissuring were handled according to the prespecified exclusion criteria; persistent cracks/fissures are also recognized in breastfeeding pain differentials within clinical guidance.^[12]^

### 2.3. Data collection

Clinical and behavioral data were retrospectively extracted from electronic medical records and standardized lactation assessment forms for breastfeeding mothers evaluated between June 2022 and 2025. The primary outcome was nipple fissures, determined using prespecified diagnostic criteria and documented at the index assessment. Data collection included 4 domains. Maternal demographic characteristics comprised age, body mass index, education level, employment status, and postpartum weight change from delivery to assessment. Obstetric and perinatal variables included parity (primiparous vs multiparous), mode of delivery (vaginal vs cesarean), gestational age at delivery, postpartum days at assessment, and preterm birth (<37 weeks). Infant characteristics included sex, birth weight, and documented tongue-tie or other oral anatomical anomalies. Feeding-related variables included feeding pattern (exclusive breastfeeding vs mixed feeding), breastfeeding frequency (sessions/day), duration per feeding session, night feeding status, latch assessment (poor latch/shallow attachment when recorded), primary feeding position (cradle, football, side-lying, or other), pain during suckling, use of feeding aids (breast pump use and pumping frequency, nipple shield use, nipple cream/ointment use), early bottle introduction (defined as bottle feeding initiated within the first 14 postpartum days prior to or at the index assessment), early pacifier use, and local breast conditions (breast engorgement, plugged ducts, inverted/flat nipples, and nipple skin dryness/desquamation). Two investigators independently verified extracted variables against source records; discrepancies were resolved by consensus with a senior reviewer. Continuous variables were recorded in original units, and categorical variables were coded using predefined categories to ensure consistency across participants.

### 2.4. Statistical analysis

All statistical analyses were performed using IBM SPSS Statistics, version 26.0 (IBM Corp., Armonk). Continuous variables were assessed for distributional characteristics and summarized as mean ± standard deviation for approximately normally distributed data or as median with interquartile range for non-normally distributed data. Between-group comparisons were conducted using the Welch *t* test for continuous variables with approximately normal distributions and the Mann–Whitney *U* test for non-normally distributed variables. Categorical variables were presented as numbers (percentage) and compared using the chi-square test; Fisher exact test was applied when expected cell counts were small. Univariate logistic regression analyses were performed to evaluate associations between candidate variables and the presence of nipple fissures, reporting odds ratios (ORs) with 95% confidence intervals (CIs). Variables considered clinically relevant and/or statistically significant in univariate analyses were entered into a multivariable logistic regression model to identify factors independently associated with nipple fissures, with results expressed as adjusted odds ratios (aORs) with 95% CIs. All statistical tests were 2-sided, and a *P* value < 0.05 was considered statistically significant. Feeding duration was modeled per 5-minute increment to improve interpretability and avoid reporting very small unit-based effect estimates. Multicollinearity was assessed using variance inflation factors, with values < 5 considered acceptable.

## 3. Results

### 3.1. Baseline demographic, obstetric, and infant characteristics

A total of 112 women met the inclusion criteria and were included in the final analysis (56 cases and 56 controls). Baseline demographic characteristics were generally comparable between groups. Maternal age did not differ significantly (29.02 ± 4.24 vs 30.11 ± 4.32 years; Welch *t* = −1.35, *P* = .181), nor did body mass index (22.99 ± 2.61 vs 23.31 ± 2.83 kg/m^2^; *t* = −0.62, *P* = .536). The median postpartum days at assessment were similar between groups (16.50 [11.19, 24.85] vs 16.87 [13.08, 24.19] days; Mann–Whitney *U* = 1500, *P* = .692). Education level (χ^2^ = 0.15, *P* = .928) and employment status (χ^2^ = 1.47, *P* = .480) showed no statistically significant between-group differences. Obstetric factors, including primiparity (60.7% vs 51.8%; χ^2^ = 0.58, *P* = .446), cesarean delivery (35.7% vs 32.1%; χ^2^ = 0.04, *P* = .842), and gestational age at delivery (39.02 ± 1.07 vs 38.83 ± 1.44 weeks; *t* = 0.81, *P* = .422), were also comparable. Infant characteristics were balanced, including birth weight (3.32 ± 0.45 vs 3.21 ± 0.50 kg; *t* = 1.29, *P* = .201), infant sex (male: 50.0% vs 60.7%; χ^2^ = 0.90, *P* = .342), and tongue-tie/oral anatomical anomalies (10.7% vs 8.9%; χ^2^ = 0.00, *P* = 1.000). Notably, postpartum weight change since delivery differed between groups (−3.99 ± 2.32 vs −2.95 ± 1.90 kg; *t* = −2.60, *P* = .011). Preterm birth occurred more frequently in the observation group (12.5% vs 1.8%); however, this difference did not reach conventional statistical significance (Fisher exact test, *P* = .061; Table 1).

**Table 1:** Baseline demographic, obstetric, and infant characteristics between groups.

Variable	Control (n = 56)	Observation (n = 56)	Test statistic	*P* value
Age, yrs	29.02 ± 4.24	30.11 ± 4.32	*t* = −1.35	.181
Body mass index, kg/m^2^	22.99 ± 2.61	23.31 ± 2.83	*t* = −0.62	.536
Postpartum weight change since delivery, kg	−3.99 ± 2.32	−2.95 ± 1.90	*t* = −2.60	.011
Gestational age at delivery, wks	39.02 ± 1.07	38.83 ± 1.44	*t* = 0.81	.422
Infant birth weight, kg	3.32 ± 0.45	3.21 ± 0.50	*t* = 1.29	.201
Postpartum days at assessment, d	16.50 (11.19, 24.85)	16.87 (13.08, 24.19)	*U* = 1500	.692
Education level			χ^2^ = 0.15	.928
High school or below	25 (44.6)	23 (41.1)		
College	29 (51.8)	31 (55.4)		
Postgraduate	2 (3.6)	2 (3.6)		
Employment status			χ^2^ = 1.47	.480
Employed	27 (48.2)	31 (55.4)		
Unemployed/housewife	24 (42.9)	18 (32.1)		
Student/other	5 (8.9)	7 (12.5)		
Primiparous, n (%)	34 (60.7)	29 (51.8)	χ^2^ = 0.58	.446
Cesarean delivery, n (%)	20 (35.7)	18 (32.1)	χ^2^ = 0.04	.842
Preterm birth (<37 weeks), n (%)	1 (1.8)	7 (12.5)	Fisher exact	.061
Male infant, n (%)	28 (50.0)	34 (60.7)	χ^2^ = 0.90	.342
Tongue-tie or oral anatomical anomaly, n (%)	6 (10.7)	5 (8.9)	χ^2^ = 0.00	1.000

BMI = body mass index, IQR = interquartile range, SD = standard deviation, *t* = Welch *t* test, *U* = Mann–Whitney *U* test, χ^2^ = chi-square test.

### 3.2. Breastfeeding practices and feeding-related factors

Exclusive breastfeeding was less frequent in the observation group than in the control group (53.6% vs 80.4%; χ^2^ = 7.91, *P* = .005). The observation group showed a shorter feeding duration (median 13.72 vs 17.51 minutes; *U* = 2204, *P* < .001), whereas daily breastfeeding frequency did not differ significantly (8.52 ± 2.43 vs 8.84 ± 1.97 sessions/day; *t* = 0.77, *P* = .443). Latch-related problems were substantially more common in the observation group, including poor latch/shallow attachment (57.1% vs 16.1%; χ^2^ = 18.62, *P* < .001) and pain during suckling (73.2% vs 26.8%; χ^2^ = 22.32, *P* < .001). The distribution of primary feeding positions was comparable between groups (χ^2^ = 4.78, *P* = .188), and night feeding rates were similar (71.4% vs 75.0%; χ^2^ = 0.05, *P* = .831). Use of breastfeeding aids was higher in the observation group, including breast pump use (60.7% vs 30.4%; χ^2^ = 9.22, *P* = .002) and a higher pumping frequency (median 1.00 vs 0.00 sessions/day; *U* = 1041, *P* < .001). The observation group also had higher rates of nipple shield use (28.6% vs 8.9%; χ^2^ = 5.86, *P* = .015), nipple cream/ointment use (58.9% vs 37.5%; χ^2^ = 4.33, *P* = .038), early bottle introduction (66.1% vs 25.0%; χ^2^ = 17.42, *P* < .001), and early pacifier use (46.4% vs 23.2%; χ^2^ = 5.66, *P* = .017). Among local breast conditions, breast engorgement (50.0% vs 26.8%; χ^2^ = 5.44, *P* = .020) and inverted/flat nipples (25.0% vs 1.8%; χ^2^ = 11.08, *P* < .001) were more common in the observation group, while plugged ducts and nipple skin dryness/desquamation were not significantly different between groups (*P* > .05; Table 2).

**Table 2:** Breastfeeding practices and feeding-related factors between groups.

Variable	Control (n = 56)	Observation (n = 56)	Test statistic	*P* value
Exclusive breastfeeding, n (%)	45 (80.4)	30 (53.6)	χ^2^ = 7.91	.005
Breastfeeding sessions per day	8.84 ± 1.97	8.52 ± 2.43	*t* = 0.77	.443
Duration per feeding session, min	17.51 [15.38, 22.15]	13.72 [10.91, 18.30]	*U* = 2204	<.001
Night feeding, n (%)	42 (75.0)	40 (71.4)	χ^2^ = 0.05	.831
Poor latch/shallow attachment, n (%)	9 (16.1)	32 (57.1)	χ^2^ = 18.62	<.001
Primary feeding position			χ^2^ = 4.78	.188
Cradle	35 (62.5)	29 (51.8)		
Football	9 (16.1)	18 (32.1)		
Side-lying	11 (19.6)	7 (12.5)		
Other	1 (1.8)	2 (3.6)		
Pain during suckling, n (%)	15 (26.8)	41 (73.2)	χ^2^ = 22.32	<.001
Breast pump use, n (%)	17 (30.4)	34 (60.7)	χ^2^ = 9.22	.002
Pump sessions per day	0.00 [0.00, 1.00]	1.00 [0.00, 2.00]	*U* = 1041	<.001
Nipple shield use, n (%)	5 (8.9)	16 (28.6)	χ^2^ = 5.86	.015
Nipple cream/ointment use, n (%)	21 (37.5)	33 (58.9)	χ^2^ = 4.33	.038
Early bottle introduction, n (%)	14 (25.0)	37 (66.1)	χ^2^ = 17.42	<.001
Early pacifier use, n (%)	13 (23.2)	26 (46.4)	χ^2^ = 5.66	.017
Breast engorgement, n (%)	15 (26.8)	28 (50.0)	χ^2^ = 5.44	.020
Plugged duct, n (%)	8 (14.3)	14 (25.0)	χ^2^ = 1.41	.234
Inverted/flat nipple, n (%)	1 (1.8)	14 (25.0)	χ^2^ = 11.08	<.001
Nipple skin dryness/desquamation, n (%)	14 (25.0)	19 (33.9)	χ^2^ = 0.69	.407

IQR = interquartile range, SD = standard deviation, *U* = Mann–Whitney *U* test, χ^2^ = chi-square test.

### 3.3. Univariate logistic regression analysis

In univariate logistic regression analyses with nipple fissures (observation group) as the dependent outcome, exclusive breastfeeding was associated with a reduced likelihood of nipple fissures (OR = 0.282, 95% CI 0.121–0.655; *P* = .003). Similarly, a longer feeding duration was inversely associated with nipple fissures, with each 5-minute increase in feeding duration corresponding to lower odds (OR = 0.601, 95% CI 0.423–0.855; *P* = .005). By contrast, latch- and pain-related variables showed positive associations with nipple fissures, including poor latch/shallow attachment (OR = 6.963, 95% CI 2.865–16.923; *P* < .001) and pain during suckling (OR = 7.471, 95% CI 3.237–17.244; *P* < .001). Several feeding aids and related practices were also associated with increased odds of nipple fissures, including breast pump use (OR = 3.545; *P* = .002), pumping frequency (OR = 1.949 per additional session/day; *P* = .002), nipple shield use (OR = 4.080; *P* = .011), and early bottle introduction (OR = 5.842; *P* < .001). Among local breast conditions, breast engorgement (OR = 2.733; *P* = .013) and inverted/flat nipples (OR = 18.333; *P* = .006) were associated with higher odds of nipple fissures. In contrast, breastfeeding frequency per day, night feeding, plugged ducts, and nipple skin dryness/desquamation were not significantly associated with nipple fissures in univariate analyses (*P* > .05; Table 3).

**Table 3:** Univariate logistic regression for breastfeeding practices and feeding-related factors.

Variable	β	SE	Wald χ^2^	OR	95% CI	*P* value
Exclusive breastfeeding (yes vs no)	−1.266	0.430	8.66	0.282	0.121–0.655	.003
Breastfeeding sessions per day (per 1 session)	−0.067	0.087	0.60	0.935	0.789–1.108	.440
Duration per feeding session (per 5-minute increase)	−0.509	0.180	8.03	0.601	0.423–0.855	.005
Night feeding (yes vs no)	−0.182	0.427	0.18	0.833	0.361–1.926	.670
Poor latch/shallow attachment (yes vs no)	1.941	0.453	18.34	6.963	2.865–16.923	<.001
Primary feeding position: football vs cradle	0.906	0.463	3.83	2.474	0.998–6.129	.050
Primary feeding position: side-lying vs cradle	−0.537	0.526	1.04	0.584	0.209–1.638	.307
Primary feeding position: other vs cradle	0.711	1.240	0.33	2.037	0.179–23.131	.566
Pain during suckling (yes vs no)	2.011	0.427	22.21	7.471	3.237–17.244	<.001
Breast pump use (yes vs no)	1.266	0.399	10.05	3.545	1.621–7.753	.002
Pump sessions per day (per 1 session)	0.667	0.210	10.06	1.949	1.290–2.944	.002
Nipple shield use (yes vs no)	1.406	0.554	6.44	4.080	1.377–12.089	.011
Nipple cream/ointment use (yes vs no)	0.872	0.387	5.07	2.391	1.119–5.108	.024
Early bottle introduction (yes vs no)	1.765	0.418	17.81	5.842	2.574–13.260	<.001
Early pacifier use (yes vs no)	1.053	0.415	6.45	2.867	1.272–6.462	.011
Breast engorgement (yes vs no)	1.006	0.403	6.22	2.733	1.240–6.023	.013
Plugged duct (yes vs no)	0.693	0.491	1.99	2.000	0.764–5.236	.158
Inverted/flat nipple (yes vs no)	2.909	1.055	7.60	18.333	2.318–145.022	.006
Nipple skin dryness/desquamation (yes vs no)	0.432	0.418	1.07	1.541	0.679–3.497	.301

CI = confidence interval, OR = odds ratio, SE = standard error, β = regression coefficient.

### 3.4. Multivariable logistic regression analysis

In the fully adjusted model, only 3 variables remained independently associated with nipple fissures. After multivariable adjustment, shorter feeding duration was independently associated with higher odds of nipple fissures. Specifically, each 5-minute increase in feeding duration was associated with a lower likelihood of nipple fissures (aOR = 0.510, 95% CI 0.301–0.863; *P* = .012). Pain during suckling remained an independent correlate of nipple fissures (aOR = 3.826, 95% CI 1.143–12.808; *P* = .030). In addition, early bottle introduction was associated with increased odds of nipple fissures after adjustment (aOR = 3.063, 95% CI 1.020–9.192; *P* = .046). In contrast, exclusive breastfeeding status, poor latch/shallow attachment, breast pump use, breast engorgement, and nipple shield use were not statistically significant in the adjusted model (*P* > .05), indicating that these variables did not demonstrate independent associations with nipple fissures when considered alongside the other covariates included in the model (Table 4).

**Table 4:** Multivariable logistic regression for breastfeeding practices and feeding-related factors.

Variable	β	SE	Wald χ^2^	aOR	95% CI	*P* value
Intercept	1.173	0.934	1.58	3.231	0.517–20.188	.208
Exclusive breastfeeding (yes vs no)	−0.725	0.538	1.82	0.484	0.169–1.392	.177
Duration per feeding session (per 5-minute increase)	−0.674	0.269	6.29	0.510	0.301–0.863	.012
Poor latch/shallow attachment (yes vs no)	0.941	0.599	2.47	2.563	0.792–8.293	.116
Pain during suckling (yes vs no)	1.342	0.617	4.73	3.826	1.143–12.808	.030
Breast pump use (yes vs no)	0.167	0.586	0.08	1.182	0.375–3.727	.775
Early bottle introduction (yes vs no)	1.119	0.561	3.98	3.063	1.020–9.192	.046
Breast engorgement (yes vs no)	0.579	0.516	1.26	1.784	0.649–4.903	.262
Nipple shield use (yes vs no)	0.789	0.636	1.54	2.202	0.632–7.669	.215

aOR = adjusted odds ratio, CI = confidence interval, SE = standard error.

## 4. Discussion

This retrospective analysis evaluated demographic, obstetric, infant, and feeding-related correlates of nipple fissures among breastfeeding mothers assessed in the early postpartum period. Baseline demographic, obstetric, and infant characteristics were broadly comparable between the nipple fissure group and controls, which reduces (but does not eliminate) the likelihood that the observed associations were driven primarily by between-group imbalance in these domains. The between-group difference in postpartum weight change suggests that maternal postpartum recovery and related behavioral or nutritional factors may differ between groups; however, the clinical interpretation of this finding is uncertain because postpartum weight dynamics can reflect multiple processes (e.g., fluid shifts, caloric intake, stress, or feeding workload), and temporality cannot be established within the present design. Feeding-related variables demonstrated clearer discrimination between groups. The nipple fissure group showed lower rates of exclusive breastfeeding, shorter feeding duration, and markedly higher frequencies of pain during suckling and latch-related difficulties. In univariate models, multiple latch-, pain-, and feeding-aid variables were strongly associated with nipple fissures, consistent with the concept that mechanical loading and friction at the nipple–areola complex are central to the development and persistence of tissue disruption and pain. Importantly, in the multivariable model, 3 factors remained independently associated with nipple fissures: shorter feeding duration, pain during suckling, and early bottle introduction. These adjusted associations suggest that the observed relationships among latch quality, pain, feeding duration, and supplementation practices are interdependent and may operate through shared causal pathways.

The inverse association between feeding duration and nipple fissures after adjustment may have several non-mutually exclusive explanations. First, shorter feeds may indicate early termination due to pain or ineffective milk transfer, which can promote suboptimal latch repetition across frequent attempts, thereby increasing localized shear forces and tissue stress. Second, shorter feeds may reflect infant frustration or compensatory sucking patterns in the context of poor attachment, increasing compressive loading on a limited area of the nipple tip rather than distributing forces across the areola. Third, feeding duration may function as a proxy for breastfeeding effectiveness, maternal confidence, or access to lactation support. Because feeding duration is modifiable, the adjusted association supports the practical value of early assessment focused on optimizing latch and effective milk transfer rather than relying solely on symptomatic treatments. Pain during suckling remained independently associated with nipple fissures and therefore merits careful interpretation. Pain is plausibly both a marker of ongoing tissue injury and a mediator that shapes feeding behaviors (e.g., shortened feeds, increased pumping, and early bottle use). Recent work emphasizes that pain intensity may be clinically consequential even when the visible extent of nipple damage is limited, and pain-based triage may identify women requiring timely breastfeeding technique support.^[4]^ In the present study, persistence of pain as an adjusted correlate indicates that pain assessment should be treated as a clinical signal rather than a secondary symptom, while recognizing that reverse causality is possible because pain may occur as a consequence of fissuring.

Early bottle introduction also remained independently associated with nipple fissures. Mechanistically, early partial bottle feeding may contribute to altered oral motor patterns, differences in suction generation, and changes in latch mechanics during subsequent breastfeeds. Evidence indicates that breast and bottle feeding involve distinct sucking and suction dynamics, supporting biological plausibility for an association between early bottle exposure and subsequent breastfeeding mechanics.^[13]^ In addition, marketing claims that bottles “prevent nipple confusion” or “aid latch” are common, whereas the empirical foundation for these claims is variable and often not clearly established, underscoring the need for cautious counseling when bottles are introduced early in the breastfeeding trajectory.^[14]^ Nonetheless, early bottle introduction may also represent a response to breastfeeding pain or perceived low milk transfer rather than a primary cause, and this bidirectionality cannot be resolved in a retrospective case-control framework. Several factors that were significant in univariate analyses were not retained as independent predictors after adjustment (e.g., poor latch/shallow attachment, breast pump use, nipple shield use, and breast engorgement). This pattern is compatible with confounding and mediation: latch problems may increase pain, which then shortens feeds and precipitates early bottle supplementation; similarly, breast pump or nipple shield use may be adopted because of pain or fissures. Studies on breast pump–related discomfort and nipple soreness suggest that technique, device settings, and fit can contribute to symptoms, but observed associations in retrospective datasets may reflect indication bias (use prompted by difficulties) rather than direct causation.^[15]^ The loss of statistical significance for inverted/flat nipples in the adjusted model should also be interpreted cautiously, because small cell counts can yield unstable estimates and wide confidence intervals, even when the crude association appears strong.

The present findings align with contemporary evidence that nipple trauma is prevalent early postpartum and is frequently linked to latch mechanics and maternal pain. A photographic image–based analysis of early nipple damage reported that nipple pain is common and clinically meaningful, and it proposed that clinical support should prioritize pain intensity rather than relying only on the apparent size or surface area of damage.^[16]^ This perspective is consistent with the current multivariable model, in which pain during suckling remained independently associated with nipple fissures and plausibly reflects an active pathophysiologic process requiring prompt breastfeeding technique assessment. A recent literature review on nipple trauma similarly summarizes latch and positioning as primary contributors to nipple injury and emphasizes the downstream consequences for breastfeeding continuation and maternal well-being.^[17]^ In addition, an evidence-based nipple care pathway has been proposed to facilitate early identification and management of breastfeeding-related nipple injuries, reinforcing the clinical utility of structured assessment of latch, pain, and feeding effectiveness rather than relying exclusively on topical or device-based solutions.^[18]^ The present data are concordant with this approach, given the strong univariate associations of shallow attachment and the persistence of pain and shortened feeds after adjustment.

With respect to supportive therapies, a recent systematic review focusing on moisturizing therapy for nipple trauma reflects ongoing interest in symptomatic management, but such interventions do not directly address mechanical drivers of tissue injury when latch dysfunction persists.^[19]^ Similarly, a 2025 meta-analysis evaluating interventions for breastfeeding-related nipple pain or injury concluded that specialized and preventive measures appear more effective than nonspecific approaches, supporting the concept that early, mechanism-directed strategies are likely to yield better outcomes.^[20]^ Regarding supplementation practices, the independent association between early bottle introduction and nipple fissures is biologically plausible in light of evidence that suction and feeding biomechanics differ between breast and bottle feeding. However, the literature also highlights that commercial bottle products frequently claim to reduce nipple confusion or support latch, while the quality and transparency of supporting evidence are inconsistent.^[14]^ Therefore, the current association should be interpreted as potentially reflecting both causal influence and reverse causation (bottle use initiated because breastfeeding has become painful or inefficient), and prospective designs are needed to clarify directionality. Finally, infant oral anatomy was balanced between groups in this study, yet contemporary evidence continues to indicate that ankyloglossia can be associated with maternal nipple pain and that frenotomy may reduce pain in some contexts, although the magnitude and certainty of benefit vary, and the risk of bias remains a concern.^[21]^ The low frequency of tongue-tie/oral anomalies in the present cohort may have limited power to detect associations, and it remains clinically appropriate to consider targeted assessment when latch dysfunction and pain persist.

This study has several limitations. First, the retrospective design introduces temporal ambiguity and precludes causal inference. Second, documentation variability may have introduced measurement error, particularly for latch assessment and pain classification. Third, the modest sample size may limit statistical power and precision, especially for low-prevalence exposures. Fourth, reverse causality cannot be excluded for several feeding-related variables. Fifth, the single-center design may limit generalizability. Finally, unmeasured psychosocial and institutional factors may contribute to residual confounding.

## 5. Conclusion

In this retrospective study, nipple fissures were primarily associated with shorter feeding duration, pain during suckling, and early bottle introduction after multivariable adjustment. Several other breastfeeding practices and local breast conditions showed significant univariate associations but did not remain independent predictors. These findings support early assessment of latch effectiveness and pain, and careful guidance on supplementation practices to reduce nipple trauma risk. These findings should be interpreted as hypothesis-generating and may inform future prospective studies designed to clarify temporality and causal pathways.

## Author contributions

**Conceptualization:** Lina Yang, Yang Li, Hui Yang, Hongmei Li, Junting Zheng, Yajing Zhang.

**Data curation:** Lina Yang, Yang Li, Hui Yang, Hongmei Li, Junting Zheng, Yajing Zhang.

**Formal analysis:** Lina Yang, Yang Li, Hui Yang, Hongmei Li, Junting Zheng, Yajing Zhang.

**Funding acquisition:** Yang Li, Yajing Zhang.

**Investigation:** Yang Li, Yajing Zhang.

**Writing – original draft:** Lina Yang, Yajing Zhang.

**Writing – review & editing:** Lina Yang, Yajing Zhang.
